# Metabolomic Profiling of Phenolic Compounds in Brazilian-Grown Olive Leaf Infusions and Ultrasound-Assisted Extracts

**DOI:** 10.3390/molecules31203740

**Published:** 2026-10-18

**Authors:** Heloisa Rodrigues Pessoa, Graziana Difonzo, Mariana Miccolis, Antonella Pasqualone, Francesco Caponio, Jerson Lima Silva, Lilia Zago, Danielly C. Ferraz da Costa

**Affiliations:** 1 Laboratory of Physiopathology and Biochemistry of Nutrition, Nutrition Institute, Rio de Janeiro State University, Rio de Janeiro 20550-013, Brazil; mrs.pessoa@gmail.com; 2 Department of Soil, Plant and Food Sciences, Food Science and Technology Unit, University of Bari Aldo Moro, Via Amendola, 165/A, I-70126 Bari, Italy; graziana.difonzo@uniba.it (G.D.); mariana.miccolis@uniba.it (M.M.); antonella.pasqualone@uniba.it (A.P.); francesco.caponio@uniba.it (F.C.); 3 National Institute of Science and Technology for Structural Biology and Bioimaging, Federal University of Rio de Janeiro, Rio de Janeiro 21941-902, Brazil; jerson@bioqmed.ufrj.br; 4 D’Or Institute for Research and Education (IDOR), Rio de Janeiro 22281-100, Brazil

**Keywords:** olive leaves, green extraction, metabolomics, multivariate analysis phenolic profile antioxidant activity

## Abstract

Olive leaves, an abundant agro-industrial by-product, represent a valuable source of phenolic compounds. Cultivars grown in Brazil remain underexplored at the metabolomic level, particularly regarding strategies for their valorization. Extraction methods, such as ultrasound-assisted extraction and infusion, offer distinct pathways for utilizing this by-product. This study aimed to characterize the phenolic metabolome (UHPLC-DAD-MS/MS) and antioxidant activity (ABTS and DPPH assays) of olive leaves from two cultivars grown in Brazil (Coratina and Picual), comparing ultrasound-assisted extracts and infusion. Multivariate analyses (principal component analysis and hierarchical clustering) were applied to evaluate sample discrimination and correlations between phenols and antioxidant activity (Spearman). The phenolic profile was complex, dominated by secoiridoids and including flavonoids, and was more efficiently recovered by ultrasound-assisted extraction. A clear distinction was observed between extraction methods and cultivars, with differences mainly attributed to the relative abundance of medium- and low-abundance metabolites. Correlation analysis indicated positive associations between multiple phenolic compounds and antioxidant activity, suggesting a synergistic effect driven by the overall metabolic profile. These findings provide novel metabolomic insights into olive leaves from Brazilian-grown cultivars and demonstrate that extraction strategy plays a key role in their valorization. Ultrasound-assisted extraction maximizes compound recovery, whereas infusions represent an accessible home-based method for enhancing antioxidant intake.

## 1. Introduction

Olive cultivation generates waste extensively and continuously, with leaves standing out among the most abundant and relatively underexplored waste streams. They result from pruning and also from the industrial stage of selecting and cleaning the fruits and olives and removing the branches and leaves [1,2,3,4]. In pruning, the leaves represent about 25% of the pruned waste [2]. Reviews provide evidence that about 10% of the mass of olives that arrive at the mill corresponds to leaves [1,3]. The abundance of this residue is an inherent consequence of the very logic of agronomic and industrial production in the olive oil chain [2,4]. On the other hand, the volume of this residue contrasts with its underutilization, as it is frequently disposed of through burning, used as energy biomass, fed to animals, or discarded, despite containing bioactive compounds. These destinations reflect the underutilization associated with economic and functional losses, in addition to environmental costs [1,2,3,4].

Olive leaves are consistently described as a rich source of phenolic compounds, predominantly secoiridoids, flavonoids, and simple phenols [2,5,6,7]. The content and distribution of phenolics depend on cultivar (genetic factor), geographical origin, leaf age, physiological state, collection season, and analytical processing conditions. Despite these changes that reflect a heterogeneous phenolic source, the evidence supports the consensus that olive leaves are a biomass rich in high-value functional compounds [5,8,9,10,11,12,13,14,15]. Thus, utilization allows for the conversion of potential environmentally impactful agro-industrial waste into an ingredient for functional foods, nutraceuticals, cosmetics, and other value-added applications. That is, in addition to waste management, the extraction of phenolics can add functions with health potential [1,3,16,17,18]. As recently highlighted by Panou and Karabagias et al. (2025), the food and beverage industry should consider olive leaf extracts to be an excellent ingredient in the development of new beverages with potential health benefits [19].

The conditions for obtaining phenolic extracts from olive leaves, such as solvent, temperature, time, process intensity, and the method itself, influence the total yield and bioactive content. That is, different extraction strategies result in distinct phenolic profiles, yields, and antioxidant activities, which implies different applications [20,21,22,23,24,25]. Ultrasound-assisted extraction has stood out as a promising technology for combining short times, efficient recovery of phenolic compounds, and a positive relationship between phenolic content and antioxidant activity [23,25,26]. It has been demonstrated that extraction by pressurized liquid and ultrasound are effective in recovering oleuropein, hydroxytyrosol, verbascoside, and flavonoids, favoring distinct profiles according to the polarity of the medium, the type of solvent used, and the intensity of the process [20,24,25,27]. Furthermore, Difonzo et al. (2017) show that ultrasound-assisted extraction is efficient using only water as a solvent, characterizing it as a green extraction method. In parallel, extraction can be performed by infusion, a home-based method that allows for consumption viability, and also can be a green extraction method using boiling water [27]. Although it has been described as a selective extraction, as it obtains part of the compounds from the matrix, less polar compounds such as triterpenes are not detected [28]. Despite this, time, temperature, cultivar, maceration, or drying of the leaves influence the composition of the infusions, showing potential for a beverage that promotes the consumption of antioxidant bioactive molecules [29,30,31].

The phenolic profile of olive leaf extracts has been extensively characterized by high-resolution chromatographic platforms coupled with mass spectrometry, revealing a complex matrix dominated by oleuropein (mainly), hydroxytyrosol, and tyrosol, but also verbascoside, rutin, luteolin and apigenin glycosides, and other minor flavonoids and phenolic derivatives. Studies using techniques such as HPLC/UHPLC–ESI-TOF/QTOF-MS demonstrate the extensive presence of phenolic compounds, ranging from 20 to 60, and show that the quantitative variation, more than qualitative, is influenced by the cultivar, season of the year, and extraction conditions [2,5,6,11,21,32,33]. The presence of these compounds is frequently associated with antioxidant activity, with consistent associations between higher phenolic content and greater scavenging or reducing capacity in assays such as DPPH, ORAC, ABTS, and FRAP [12,13].

Moreover, there is a growing use of metabolomics with multivariate analyses to discriminate cultivars, track chemical markers, and understand how origin and method modulate the profile of olive leaf extracts [14,34,35]. The literature that details the identification and quantification of phenolic profiles, especially chemometrics, focuses on olive trees cultivated in Spain, Italy, Greece, Tunisia, Morocco, and China [8,11,13,32,34,36]. While Brazilian olive cultivation remains less explored, with studies focusing on seasonal variation in phenolic profiles and green extraction [37,38,39], there are still few studies that explore the composition of cultivars influenced by these characteristics. In this context, metabolomic approaches emerge as promising tools to integrate these dimensions and enhance the understanding of the technological and health potential of the phenolic profile of olive leaves as an agro-industrial residue.

This study aimed to explore the valorization of an underused agro-industrial waste in Brazil, that is, olive leaves from two cultivars (Coratina and Picual), using extraction via ultrasonic and infusion methods. It involved a thorough characterization of the metabolomic profile of phenolic compounds, including their identification and quantification, as well as an assessment of their antioxidant capacity. This evaluation sought to determine their potential as a source of value-added secondary metabolites.

## 2. Results and Discussion

### 2.1. Analysis of Phenolic Metabolites by UHPLC-DAD-MS/MS

The phenolic compounds were identified based on the analysis of each peak generated in the chromatography, considering retention times, corresponding wavelengths (240, 280, 335, 350 nm), precise mass measurements, and ion fragmentation. Identification of phenolic metabolites was supported by comparison with literature-reported MS/MS profiles, enabling the systematic characterization of the main secondary metabolites present in each cultivar and extraction method.

Table 1 and Table 2 provide a detailed identification of phenolic metabolites in olive leaf extracts (OLE) and olive leaf infusions (OLI), respectively. Each table includes retention times, molecular ions ([M–H]^−^), UV maxima, and main MS/MS fragments for all compounds detected in each cultivar and extraction method.

UHPLC-MS-based profiling enabled the annotation of phenolic metabolites through the combined interpretation of accurate mass, retention behavior under the chromatographic conditions, UV data, MS/MS fragmentation, and retention time, providing complementary support for distinguishing compounds within the same analytical method. In agreement with the literature, MS/MS information was particularly useful for strengthening the assignment of isomeric or structurally related metabolites with similar *m*/*z* values [21,45,46,47]. This analytical strategy allowed a broader visualization of the phenolic chemical space recovered by each extraction protocol.

This broader analytical resolution is particularly relevant in the Brazilian context, where studies on olive leaves have mainly focused on extraction performance, antioxidant activity, key phenolic compounds such as oleuropein, and a wider metabolic variation [37,38,48,49,50]. Thus, our results expand the current Brazilian literature, showing, with greater analytical detail, how the cultivar and extraction protocol jointly determine the recovered phenolic profile.

**Figure 1:**
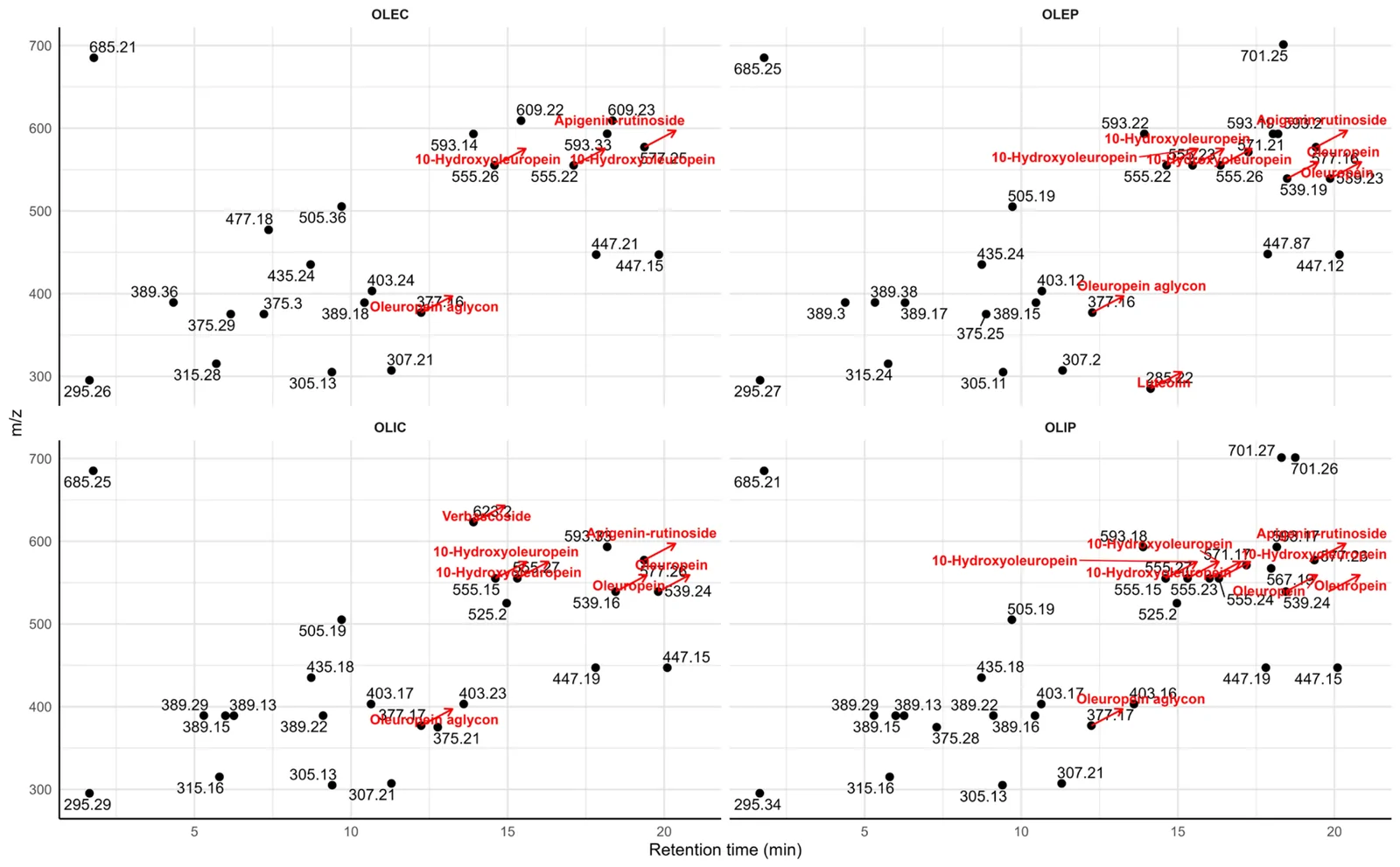
RT–*m*/*z* distribution of phenolic metabolites detected in olive leaf extracts and infusions, complementing the detailed profiles presented in Table 1 and Table 2. Selected metabolites are highlighted in red to emphasize key differential markers among extraction methods and cultivars. OLEC: olive leaf extract (ultrasound-assisted) from Coratina; OLIC: olive leaf infusion from Coratina; OLEP: olive leaf extract (ultrasound-assisted) from Picual; OLIP: olive leaf infusion from Picual.

In particular, twenty-one compounds were identified in OLE samples, whereas nineteen molecules were reported for OLI samples. Secoiridoids, flavonoids, hydroxycinnamic acid derivatives, phenylethanoid glycoside and flavanols were identified. As shown in Table 1, the OLE of the two cultivars analyzed showed different phenolic metabolites; Coratina leaves showed a phenolic profile more abundant in flavonoids (rutin, quercetin-O-rhamnoside, luteolin-dihexoside), compared to leaves from Picual cultivar, which were marked by compounds of secoiridoid class (elenolic acid dihexoside, oleuropeinic acid, oleuropein-di-O-hexoside, oleuropein-O-hexoside). Elenolic acid dihexoside and rutin of OLI showed a similar trend, being identified in Picual and Coratina cultivar, respectively (Table 2). Conversely, oleuropeinic acid and oleuropein-di-O-hexoside, exhibited an opposite trend, compared to OLE, being identified in Coratina sample. In line with this pattern, verbascoside was reported in Picual cultivar following infusion extraction. The extraction method was associated with marked differences in the phenolic profile, depending on the cultivar. For instance, ultrasound-assisted extraction of the Coratina sample resulted in a profile characterized by a greater representation of flavonoids, whereas secoiridoids were more prominently represented in the infusion profile. As regards Picual cultivar, just demethyl-oleuropein showed the same trend; whereas oleuropein-O-hexoside and oleuropeinic acid were identified exclusively after ultrasound-assisted extraction. These baseline profile differences are further explored in Figure 1.

To further investigate the distribution of phenolic compounds across the chemical space, RT–*m*/*z* scatter plots were constructed (Figure 1). These plots illustrate the dispersion of metabolites according to their retention time and mass-to-charge ratio, reflecting differences in polarity and molecular weight. A broad distribution of compounds was observed across all samples, indicating high chemical diversity. The compounds were spread out along the retention time axis based on their polarity. More polar phenolics came out first, while less polar, more structurally complex secoiridoids came out later in the chromatographic run. Compounds such as oleuropein and its derivatives, including 10-hydroxyoleuropein and oleuropein aglycon, were predominantly located in regions of higher *m*/*z* and longer retention times, reflecting their lower polarity and greater molecular complexity. In contrast, simpler phenolic compounds, such as hydroxytyrosol derivatives and phenolic acids, were observed at lower *m*/*z* values and shorter retention times. This distribution complements the PCA results by providing a spatial representation of metabolite diversity and reinforcing the contribution of specific phenolic classes to sample differentiation.

In reversed-phase liquid chromatography (C18), retention behavior is primarily influenced by hydrophobic interactions, with more polar compounds generally eluting earlier and less polar compounds exhibiting longer retention times. However, this relationship is not absolute and depends on structural factors such as glycosylation, hydroxylation, ionization state, and molecular size. Therefore, comparisons of retention time are more reliable within the same class of phenolic compounds and should be interpreted alongside *m*/*z* values and MS/MS fragmentation patterns [21,45,51,52,53,54].

Extracts (OLE) exhibited a greater presence of high-molecular-weight secoiridoids distributed in specific retention time regions, whereas infusions (OLI) showed a comparatively simplified profile, suggesting possible degradation and/or lower recovery of complex phenolics. Among the highlighted metabolites, compounds such as oleuropein, oleuropein derivatives (oleuropein aglycone and diglucoside forms), elenolic acid derivatives, and flavonoids, including luteolin and apigenin glycosides, emerged as key contributors to the differentiation between experimental groups.

Differences between extraction methods in the same cultivar can be noted. OLEC presents phenolics with *m*/*z* 609 (luteolin-diglucoside and rutin) and *m*/*z* 477 (luteolin-O-hexoside). This indicates that the ultrasound-assisted extraction method was efficient for glycosylated flavonoids (more polar, as seen in the graph). The predominance of these compounds in regions of shorter retention time reinforces the method’s affinity for highly hydrophilic metabolites (lower tR, elute first) [51,52]. While OLIC does not show the same profile, particularly the compounds with *m*/*z* 609 and *m*/*z* 477 detected in OLEC. However, luteolin glucoside (*m*/*z* 447) was detected in both samples. This pattern suggests a more selective and less comprehensive extraction in terms of the diversity of glycosylated flavonoids, which are known to exhibit reduced retention in reverse-phase systems due to the increased polarity associated with the degree of glycosylation [21,55,56].

OLEP shows the emergence of luteolin (*m*/*z* 285) in its free form (aglycone), which is less polar (RT ~14 min), consistent with the greater retention usually observed for aglycones compared to their glycosylated molecules [21,55]. This suggests that the ultrasound-assisted extraction method in the Picual cultivar seems to have caused hydrolysis or was more selective for less complex forms, which was not observed in the infusion extraction. In contrast, OLIP showed the presence of compounds with distinct chromatographic behavior, including elenolic acid dihexoside (*m*/*z* 567.19; RT 17.98 min), illustrating the diversity of phenolic compounds detected in the Picual infusion.

Common to both extractions are the compounds *m*/*z* 701 (oleuropein-O-hexoside) and *m*/*z* 571 (oleuropeinic acid), which are compounds more associated with the Picual cultivar compared to the Coratina cultivar, whether by ultrasound-assisted extraction or infusion. Specifically, the presence of *m*/*z* 701 (oleuropein-O-hexoside) in both methods is an indicator of extraction efficiency for complex high-polarity secoiridoids, which may be associated with the genetics of the Picual cultivar. The location of these compounds in regions of higher *m*/*z* and intermediate to high retention reflects not only their structural complexity but also the combined influence of hydrophobicity, molecular size, and possible ionization states on chromatographic retention [45,53,57]. Furthermore, both infusions are marked by the presence of *m*/*z* 525 (demethyl-oleuropein). This compound, distributed in intermediate retention regions, may indicate partial transformations of secoiridoids during the aqueous extraction process, as oleuropein derivatives, such as demethylated forms, have been described in olive leaf profiles by LC-MS [40], and its chromatographic retention may reflect structural modifications that affect polarity and interactions with the stationary phase [54].

Table 3 summarizes the concentrations of phenolic metabolites identified in both OLE and OLI for the two olive leaf cultivars. Values are expressed as mg 100 g^−1^ of dry leaves (mean ± SD, *n* = 3), allowing direct comparison of the phenolic profiles across extraction methods and cultivars.

The phenolic composition of olive leaves was largely dominated by secoiridoids, with a highlight on oleoside, which represented more than 50% of the total content in all samples (for example, 861.01 mg/100 g; 55.5% in OLEC and 512.42 mg/100 g; 59.5% in OLIC). Other relevant secoiridoids included elenolic acid glucoside (7.9–11.7%), oleuropein (6.5–10.1%), and 10-hydroxyoleuropein (5.3–6.2%), which together constitute the dominant core of the phenolic profile.

Diverging from most of the literature that frequently addresses oleuropein as the major compound, the presence of oleoside as the major compound in the present study may be associated with factors such as cultivar, cultivation region, climatic conditions, and seasonality, which directly influence the phenolic profile of olive leaves [58]. The presence of oleuropein may have an inverse relationship with derivatives resulting from the breakdown of its molecule [40]; consistently, elenolic acid glucoside showed a slightly higher contribution than oleuropein. While in Portuguese cultivars Negrinha do Freixo and Cornicabra, secologanoside (an isomer of oleoside) was described as one of the major constituents, along with the occurrence of elenolic acid diglucoside and oleuropein aglycone, also detected in the samples of this study [59]. Methanolic extracts of the Arbequina, Picual, and Manzanilla cultivars from the southern region of Brazil were characterized and, like our findings, did not present oleuropein as the major compound. However, their profile was predominantly dominated by flavonoids [38].

Moreover, the presence of oleuropein not as a major compound can favor the acceptability of the consumption of infusions, according to the findings of Vieitez et al. (2026), who observed that infusions of the Arbequina cultivar with higher oleuropein content intensify sensory attributes such as bitterness, astringency, and green flavor and aroma, which were perceived as unpleasant by most consumers, and infusions with milder characteristics of these same attributes are more likely to be accepted and purchased by consumers, according to the sensory analysis conducted [60].

In our findings, the differences between the extraction methods were more evident in the flavonoid fraction. The extracts obtained by ultrasound showed higher concentrations of luteolin glucoside (up to 125.3 mg/100 g; 7.9% in OLEP) and apigenin-O-rutinoside (58.56 mg/100 g; 3.7% in OLEP), while these compounds were reduced in infusions (≈3–4% and ≈2%, respectively). Corroborating with Preczenhak et al. (2025), which showed that in infusions of Arbequina olive leaves, cultivated in the Brazilian southeast, flavonoids were the main compounds affected by the boiling process [48].

Our results are consistent with previous studies, which widely recognize olive leaves as rich sources of phenolic compounds, particularly secoiridoids, flavonoids, and phenylethanoid derivatives, which have been consistently identified in different cultivars and extraction methods [33,34,48].

The total amount of phenolic compounds extracted varied significantly between the extraction methods, highlighting a marked effect of the technique employed. The extracts obtained by ultrasound showed higher yields (1552.0 and 1593.8 mg/100 g for OLEC and OLEP, respectively), while the infusions yielded about 50% less (861.0 and 868.6 mg/100 g for OLIC and OLIP). Difonzo et al. (2022) reported similar yield ranges in extracts from Italian olive leaves obtained by ultrasound (water:ethanol, 70:30 *v*/*v*), expressed in mg/100 g of dry leaves, for the cultivars Bambina (1349.1 to 1816.7), Cima di Melfi (1241.4 to 1542.3), and Leccino (928.4 to 1681.0), including the Coratina cultivar (1733.0 to 1742.6). Medina et al. (2019), in their study, corroborate our findings by analyzing a range of leaf samples, extracts, and infusions and demonstrating that less than 50% of the leaf polyphenols are recovered in the aqueous phase during infusion [28,34].

Ultrasound-assisted extraction and infusion are techniques with distinct principles and objectives. In ultrasound-assisted extraction, it is strengthened by the principle of cavitation and cell rupture. Ultrasonic waves generate bubbles that collapse in the liquid medium, forming micro-jets and micro-currents capable of breaking superficial structures of the plant matrix, increasing solvent penetration, and accelerating mass transfer [3,16,61]. Clodoveo et al. (2021) highlighted it as a green technology that optimizes the extraction of olive leaf extracts, compatible with the use of water as a solvent, with reduced time and lower thermal degradation. Difonzo et al. (2017) compared the use of alcoholic and aqueous solvents in different proportions and demonstrated the efficiency of recovery using only an aqueous solvent through the ultrasound-assisted extraction method [3,27]. Infusion, on the other hand, is a simple aqueous extraction intended for consumption, based on diffusion into water under beverage-compatible preparation conditions, and therefore tends to be more selective and less exhaustive. The extraction depends on the hydration of the matrix, solubilization in water, and diffusion of the compounds into the liquid phase over time, with a strong dependence on temperature, particle size, and contact time [28,29,30,31]. In addition to the increase in temperature, which usually reaches the boiling point, this process is associated with an enhanced transfer of compounds to the water [29,30]. Overall, infusion is associated with the recovery of more hydrosoluble and easily diffusible compounds, while less soluble compounds or those more retained in the matrix are under-extracted or not detected [28,30]. Regarding the application objectives of these methods, the ultrasound method has been associated with olive leaf extracts for nutraceutical, functional, and by-product valorization applications [23,25,62], while infusion is intended for consumption and for domestic or commercial use as functional tea [29,31].

Our results are in agreement with the literature that has already demonstrated that extracts obtained by UAE tend to concentrate more phenolics per mass of extract or leaf application [23,25,62], while infusions are more diluted and depend on the volume of water, granulation, and contact time [29,31]. Additionally, it is evident that the extraction system not only affects the yield but also qualitatively modulates the recovered phenolic profile. In this context, the data obtained for leaves cultivated in the Serra da Mantiqueira expand the geographical representativeness of Brazilian studies, indicating that, despite quantitative and relative variations among compounds, the main classes of phenolics described for the species remain conserved.

The drying and extraction procedures represent potential limitations of the present study and should be considered when interpreting these profiles. Although the drying condition of 120 °C for 15 min was adopted from previously reported procedures for olive leaves by Difonzo et al. (2017) and Pessoa et al. (2026), the relatively high temperature may promote partial degradation or transformation of heat-sensitive phenolic compounds. Moreover, differences in solvent-to-solid ratio, temperature, and extraction time (UAE: 1:20, room temperature, three 30-min cycles; AIE: 1:140, boiling water, 5 min) may influence the recovery of individual phenolic compounds [27,39]. Therefore, the profiles reported herein represent the dried material and specific extraction conditions employed, rather than the complete phenolic composition of fresh leaves.

### 2.2. Multivariate Analysis and Key Differential Phenolic Metabolites and Abundance Patterns

**Figure 2:**
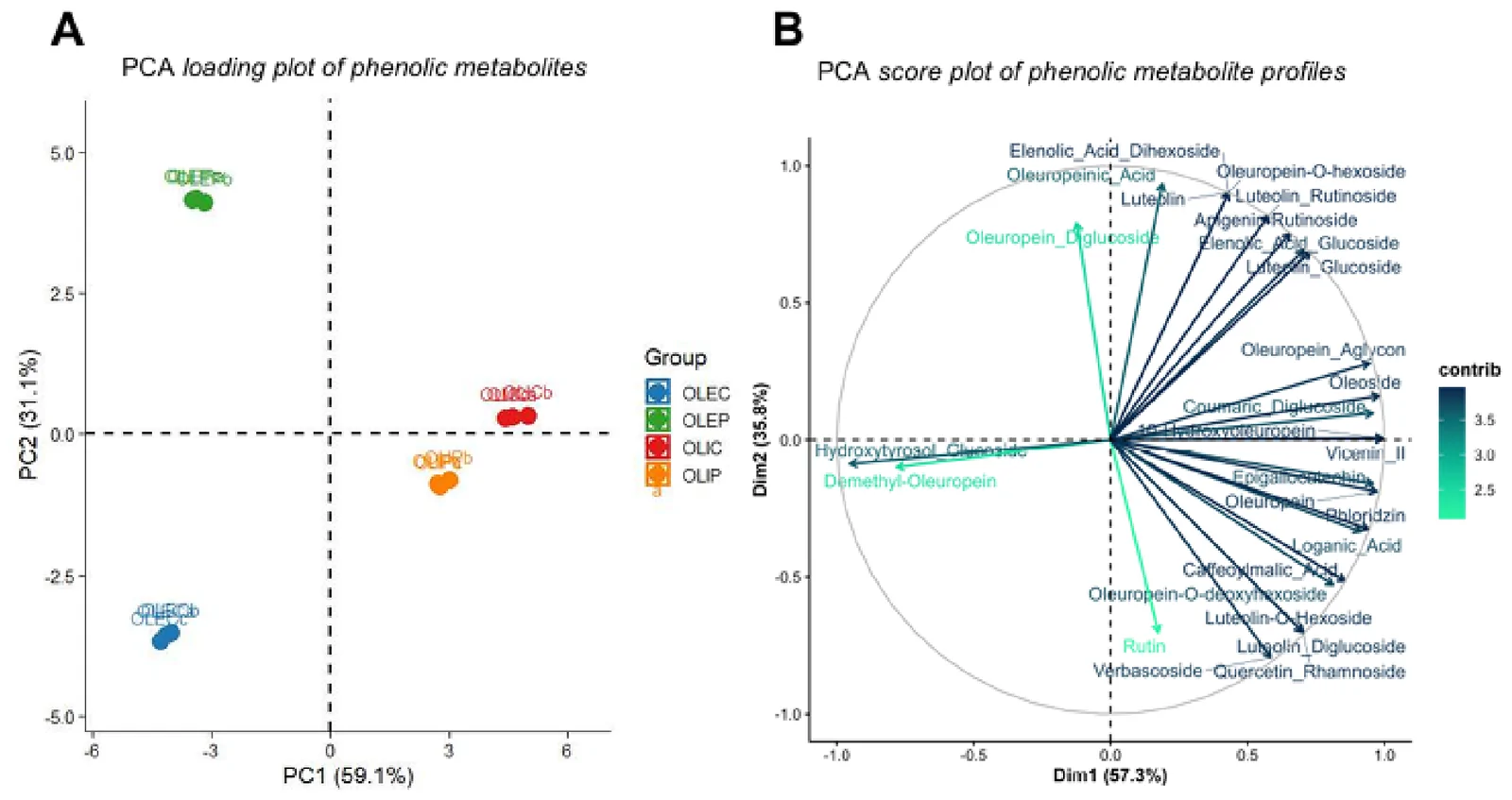
Multivariate analysis and chemical space distribution of phenolic metabolites. (**A**) PCA score plot showing the discrimination between extracts (OLE) and infusions (OLI), as well as between Coratina (C) and Picual (P) cultivars. (**B**) PCA loading plot indicating the metabolites contributing to sample separation. OLEC: olive leaf extract (ultrasound-assisted) from Coratina; OLIC: olive leaf infusion from Coratina; OLEP: olive leaf extract (ultrasound-assisted) from Picual; OLIP: olive leaf infusion from Picual.

Multivariate analysis was employed to explore the phenolic metabolite profiles of olive leaf extracts and infusions obtained from Brazilian cultivars (Figure 2).

Principal component analysis (PCA) showed a clear difference between the experimental groups (Figure 2A). The first two principal components accounted for a significant portion of the total variance (PC1: 59.1%, PC2: 31.1%), indicating that most of the variation in the phenolic dataset was captured by these two dimensions. The score plot also showed separation according to both extraction procedure and cultivar, indicating that these factors contributed to differences in the overall phenolic profiles. Notably, the ultrasound-assisted extracts (OLEC and OLEP) showed a more pronounced separation between cultivars, whereas the infusion samples (OLIC and OLIP) occupied closer positions in the score plot. Despite a broadly similar qualitative composition dominated by major secoiridoids across samples, the observed separation reflects differences in the relative distribution of both major and lower-abundance metabolites across the experimental groups.

The more pronounced separation between cultivars observed in the ultrasound-assisted extracts may reflect differences in the extraction behavior of phenolic metabolites. Ultrasound-induced acoustic cavitation can enhance matrix disruption and mass transfer, potentially facilitating the release of phenolic compounds and making cultivar-related differences in their abundance more apparent. In contrast, hot-water infusion may favor the extraction of metabolites that are readily released and soluble under the preparation conditions, while boiling may promote the degradation of some compounds, resulting in more similar profiles for certain metabolites [23,30,61,62].

The loading plot (Figure 2B) shows which metabolites contribute to the separation of the samples. Compounds positioned in the same direction as the sample clusters indicate metabolites associated with the respective groups. Samples from OLEP were associated with elenolic acid dihexoside, oleuropeinic acid, and oleuropein diglucoside, whereas OLEC samples were more closely associated with hydroxytyrosol glucoside and demethyl-oleuropein. Among the infusion samples, OLIC was positioned in the direction of oleuropein aglycon, oleoside, and coumaric acid diglucoside, while OLIP showed a closer association with rutin, verbascoside, quercetin rhamnoside, and luteolin diglucoside. Other metabolites, including oleuropein-O-hexoside, luteolin rutinoside, and apigenin rutinoside, contributed to the positive PC1/PC2 region and further differentiated the experimental groups. Metabolites located farther from the origin contributed more strongly to the variance represented by the respective principal components and may therefore represent important variables underlying the observed separation pattern.

**Figure 3:**
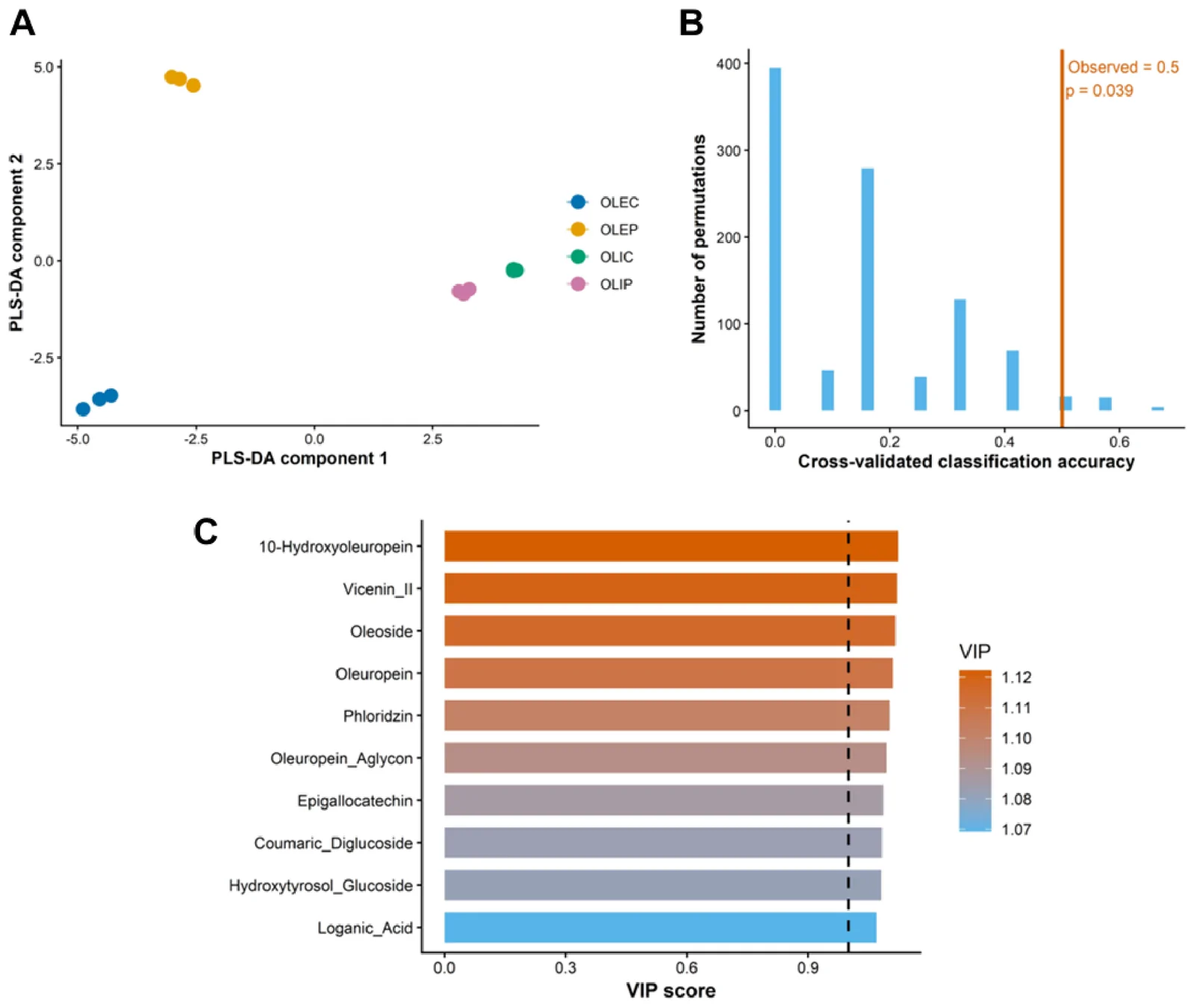
Validation of the PCA model and supervised PLS-DA of phenolic metabolites. (**A**) PLS-DA score plot showing the distribution of OLEC, OLEP, OLIC, and OLIP samples according to the first two latent components. (**B**) Permutation test of the PLS-DA model based on cross-validated classification accuracy after 1000 random class-label permutations; the vertical line indicates the observed model performance, and the permutation-derived *p*-value is shown. (**C**) Variable importance in projection (VIP) scores for the ten metabolites with the highest contribution to group discrimination; the dashed line indicates the conventional VIP threshold of 1.0.

Overall, the PCA indicates that the differentiation among experimental groups is associated with multicomponent phenolic signatures, with different subsets of secoiridoids, flavonoids, and other phenolic metabolites contributing to the separation of the extraction methods and cultivars. The PCA model validation results, including cumulative R^2^X and Q^2^X across the principal components, are provided in Supplementary Figure S1. The PCA model showed that the first two components accounted for 93.9% of the cumulative variance (R^2^X), while the corresponding cumulative Q^2^X reached 0.92, indicating that the main structure captured by the model was consistently reproduced during cross-validation. To further evaluate the discriminatory contribution of individual metabolites, supervised partial least squares discriminant analysis (PLS-DA) was subsequently performed (Figure 3).

The supervised PLS-DA further showed separation among the four sample groups, with OLEC, OLEP, OLIC, and OLIP occupying distinct regions of the score space (Figure 3A). The observed cross-validated classification accuracy was 0.50, and the permutation test based on 1000 random class-label permutations yielded a *p*-value of 0.039, indicating that the observed classification performance was significantly different from that obtained under random class assignment (Figure 3B). The VIP analysis identified ten metabolites with VIP values above 1.0, indicating their greater contribution to the discrimination among the sample groups (Figure 3C). These metabolites included elenolic acid dihexoside, oleuropein-O-hexoside, luteolin, verbascoside, oleuropeinic acid, quercetin rhamnoside, luteolin diglucoside, luteolin-O-hexoside, luteolin rutinoside, and oleuropein diglucoside.

**Figure 4:**
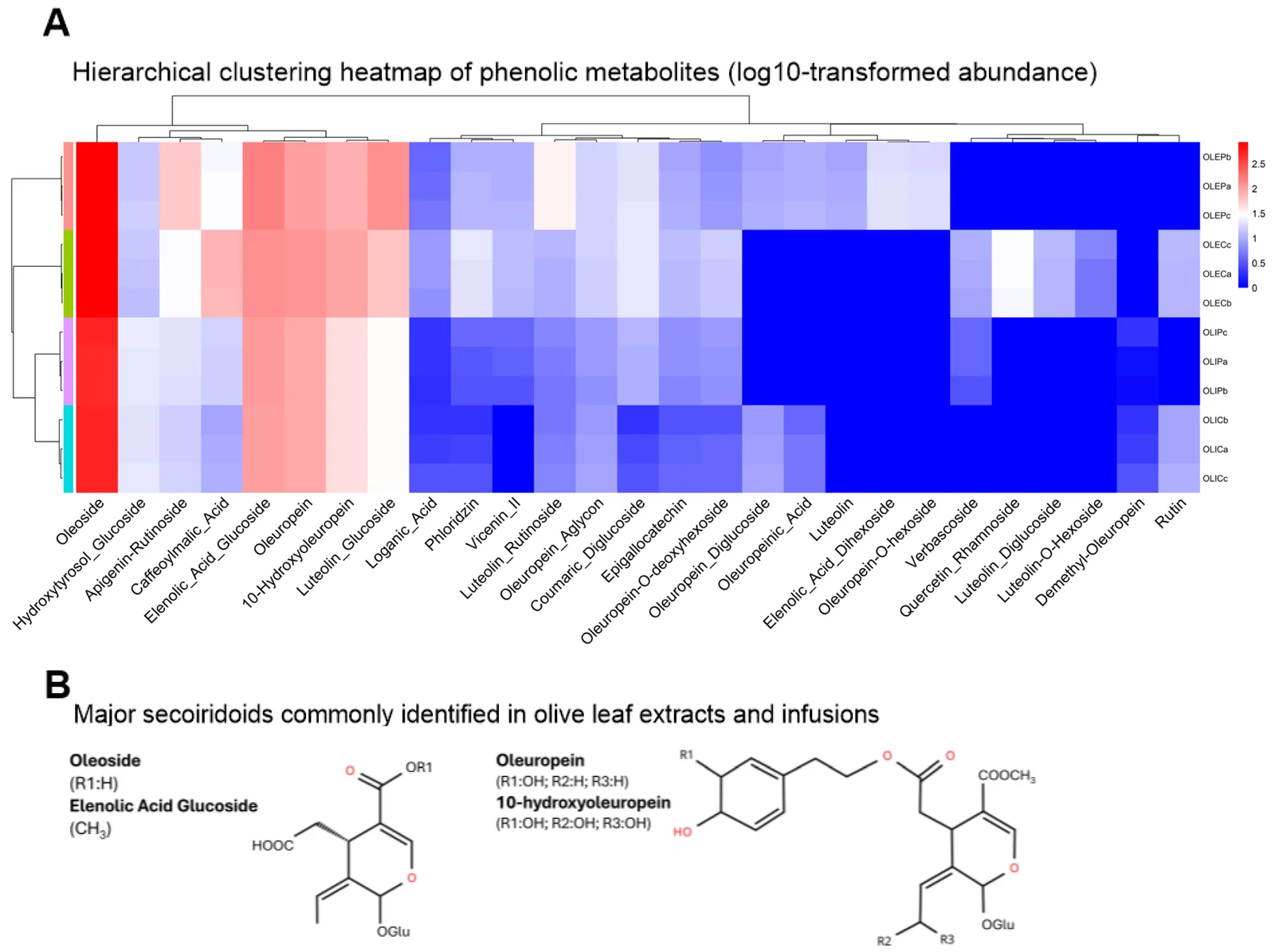
Differential analysis and abundance patterns of phenolic metabolites in olive leaf samples. (**A**) Heatmap of log_10_-transformed phenolic metabolite abundances across the 12 individual samples, with hierarchical clustering of samples and metabolites. (**B**) Representative structures of major secoiridoids detected in olive leaf extracts and infusions, including oleoside, elenolic acid glucoside, oleuropein, and 10-hydroxyoleuropein. OLEC: olive leaf extract (ultrasound-assisted) from Coratina; OLIC: olive leaf infusion from Coratina; OLEP: olive leaf extract (ultrasound-assisted) from Picual; OLIP: olive leaf infusion from Picual.

Hierarchical clustering analysis was subsequently performed as a complementary unsupervised approach to PCA, allowing the similarity among individual samples and their phenolic abundance patterns to be examined (Figure 4A).

The heatmap based on log_10_-transformed relative abundances (Figure 4A) revealed that the individual samples clustered predominantly according to the extraction method with the three replicates of each experimental group showing similar abundance patterns and, to a lesser extent, according to the cultivar, corroborating the patterns observed in the PCA. The hierarchical clustering of metabolites (dendrogram) revealed the formation of clusters with similar abundance patterns, indicating co-variation among specific subsets of phenolic compounds. These metabolite clusters comprised compounds with similar distribution across the experimental groups, including groups enriched in secoiridoids and others containing flavonoid derivatives.

The hierarchical clustering analysis of phenolic metabolites revealed distinct abundance patterns associated with the extraction method and cultivar. Extracts obtained by ultrasound (OLEC and OLEP) generally showed higher relative abundances of several secoiridoids and glycosylated flavonoids than the infusions (OLIC and OLIP). Among the metabolites showing marked differences across the experimental groups, 10-hydroxyoleuropein, luteolin glucoside, apigenin-rutinoside, and caffeoylmalic acid showed higher relative abundances in the ultrasound-assisted extracts. At the cultivar level, Coratina samples showed a relatively higher abundance of some secoiridoid derivatives, including oleuropein and its related forms, verbascoside, quercetin derivatives, and luteolin derivatives, confirming their well-known richness in phenolic compounds. In contrast, Picual samples exhibited relatively higher abundances of several luteolin derivatives and apigenin-rutinoside, as well as simpler phenolic compounds, such as hydroxytyrosol glycoside. These patterns are consistent with the separation observed in the PCA.

The observed abundance and clustering patterns are broadly consistent with previous studies describing secoiridoid fraction as metabolically more conserved in olive leaves, whereas flavonoids and minor phenolics respond more dynamically to extraction and processing conditions [28,38,40].

Metabolites of medium and low abundance exhibited greater variability, playing a relevant role in the differentiation of groups, whereas major compounds, such as oleuropein-derived secoiridoids, displayed more moderate variations in their relative abundances, also contributing to the characterization of metabolic profiles. This pattern suggests a distinction between compounds that define the chemical identity of the matrix (major compounds) and those that modulate statistical discrimination among groups (minor compounds), reinforcing the role of the latter as sensitive markers to processing. The presence of the flavonoid luteolin glucoside among the most abundant compounds, specifically in ultrasound-assisted extracts (OLE), corroborates the separation of groups and indicates that the extraction method selectively influences the recovery of this class. This is likely due to increased cell permeabilization and intensification of cavitation phenomena, already associated with higher efficiency in the extraction of more polar phenolic compounds [14,63].

Among the major phenolics (Figure 4B), oleoside stood out as the most abundant metabolite in all samples, followed by elenolic acid glucoside, with a consistent presence of oleuropein and 10-hydroxyoleuropein. This metabolic profile reinforces the existence of a conserved phenolic core in olive leaves, widely reported in the literature across different cultivars and extraction conditions [28,38,64]. The conservation of this metabolic profile has been associated with the biosynthetic stability of the secoiridoid pathway, derived from mevalonic acid/iridoid metabolism, which confers relative consistency of this group against external variations [34].

Although this central metabolic signature is maintained, the quantitative variations observed among samples highlight that extraction processing acts as a determining factor in modulating the phenolic profile, particularly in the redistribution of minor compounds and flavonoids. In this sense, the observed differences do not only reflect variations in matrix composition but also the fraction of the metabolome that becomes accessible under different extraction conditions, indicating that the detected profile is, in part, dependent on the extraction strategy employed. In this context, recent studies indicate that green techniques may not only increase overall yield but also selectively alter the relative composition of extracts, favoring compounds with greater structural accessibility or differential solubility [14,19,63]. Additionally, agronomic factors such as cultivar, harvest period, and edaphoclimatic conditions of southeastern Brazil may contribute to the observed variability, as it has already been demonstrated that environmental stresses and cultivation conditions significantly modulate secondary metabolism in olive leaves [34,40].

In general, the results indicate that, despite the conservation of a central phenolic signature dominated by secoiridoids, green extraction methods, especially ultrasound-assisted extraction, play a crucial role in redefining the detected metabolic profile, not only in terms of yield but also in the amplification of lower-abundance discriminant metabolites.

Ultrasound-assisted extraction showed a higher global abundance of phenolic compounds, which is consistent with the cavitation effect and enhanced disruption of the plant matrix described in the literature [27,34]. Infusions, on the other hand, exhibited a distinct set of minor metabolites, despite the lower overall yield. This behavior is consistent with the fact that aqueous extraction favors more polar and readily soluble compounds, transferring only a fraction of the total phenolics from the matrix, while retaining key compounds such as oleuropein derivatives, thus enabling their consumption [28,29]. Thus, the distinction between overall yield and extractive selectivity becomes essential for the interpretation of metabolomic profiles and for the valorization of different approaches in the utilization of olive leaves.

In agreement with metabolomic studies on olive leaves, the discrimination among samples was primarily driven by multicomponent chemical signatures, rather than by isolated variations in a single metabolite [8,14,34,40]. Profiles enriched in luteolin derivatives, rutin, and other flavonoids are consistent with flavonoid-dominated profiles previously described for the differentiation among cultivars and cultivation conditions [12,13,14,34], whereas profiles enriched in oleuropein and its derivatives correspond to the secoiridoid axis classically reported for olive leaves [5,40,65,66]. In this scenario, the differences between ultrasound and infusion primarily reflect the selectivity of extraction processes, with a relative shift between more flavonoid-enriched profiles and profiles more centered on secoiridoids.

Overall, in this study, flavonoids contributed more prominently to the differentiation of ultrasound-assisted extracts, whereas infusion profiles were more strongly associated with secoiridoids and their derivatives, reflecting differences in the selectivity of the extraction processes. This pattern is aligned with previous studies on olive leaves, in which the differentiation among cultivars and environmental conditions is not driven by major compounds such as oleuropein but rather by relative variations in secondary secoiridoids, hydroxytyrosol derivatives, and flavonoids, particularly luteolin-derived compounds and verbascoside [12,13,34,40,67].

Luteolin-derived flavonoids have been described as robust markers of variability among cultivars, environmental conditions, and analytical strategies [13,14,47,67]; whereas compounds such as quercetin rhamnoside often exhibit a more sporadic occurrence and genotype-dependent distribution, being more frequently described as specific discriminant markers rather than a central component of the phenolic profile [12,13,34].

In summary, these results indicate that the differences between methods are not limited to extraction efficiency but rather reflect the recovery of complementary chemical fractions of the leaf metabolome. Thus, while ultrasound enhances yield and alters the composition of detected metabolites, infusion preserves a more restricted profile, yet one that is representative of consumption conditions, highlighting that both methods are complementary in the characterization of the phenolic profile.

### 2.3. Integrated Analysis of Antioxidant Activity and Phenolic Profile in Olive Leaf Extracts

Antioxidant activity of olive leaf extracts and infusions was evaluated using DPPH and ABTS radical scavenging assays, and the results were further explored through Spearman correlation analysis with the phenolic profile. Figure 4 presents both the antioxidant activity of the extracts and the corresponding correlation matrix, highlighting the relationships between phenolic compounds and radical scavenging capacity.

**Figure 5:**
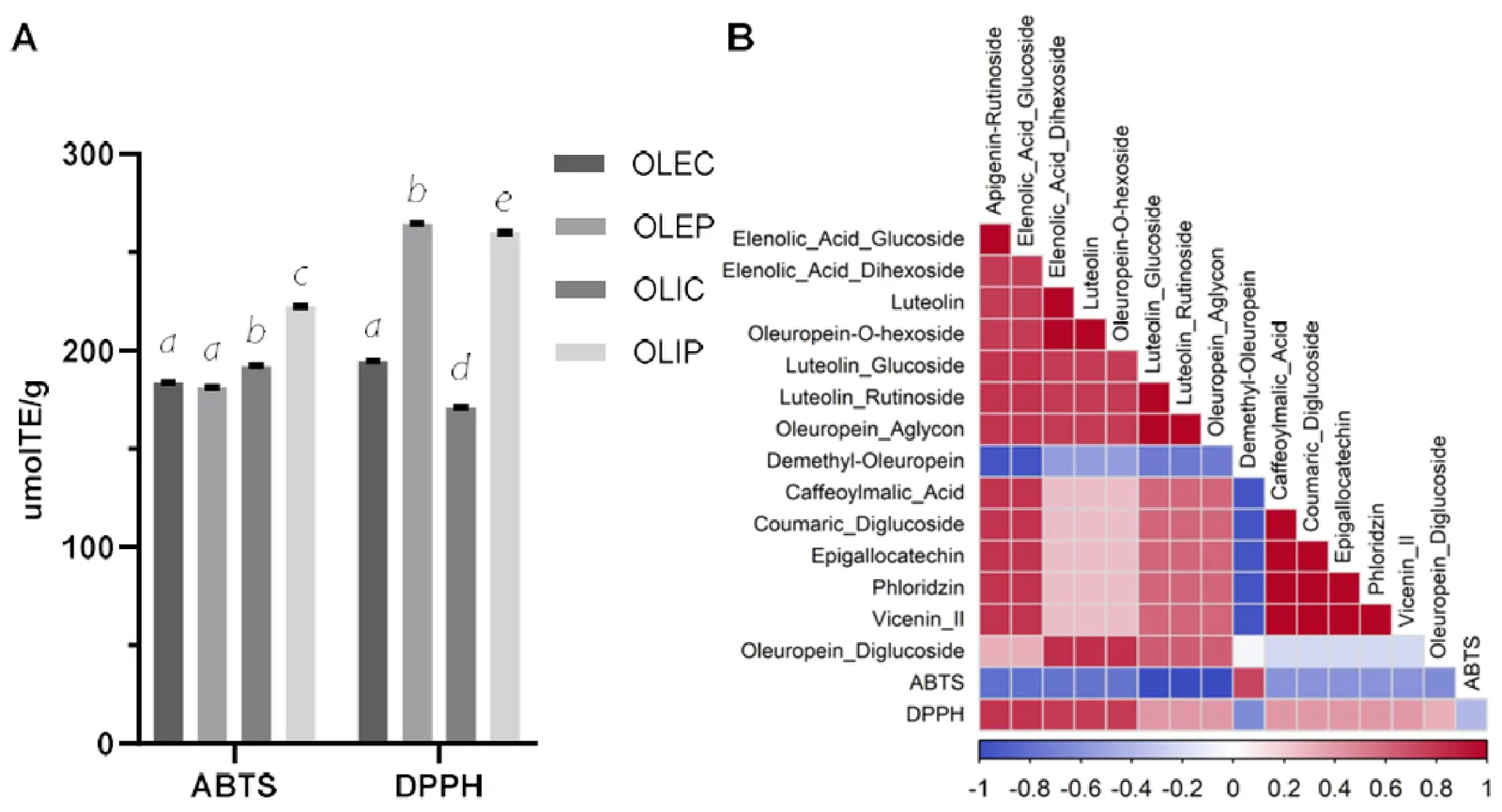
Antioxidant activity and correlation analysis of phenolic compounds in olive leaf extracts. (**A**) Antioxidant activity determined by ABTS and DPPH assays. Data are expressed as mean ± standard deviation (*n* = 3). Different letters indicate significant differences between samples (one-way ANOVA followed by Tukey’s test, *p* < 0.0001). (**B**) Spearman correlation matrix showing associations between major phenolic compounds and antioxidant activity. The matrix is displayed as a lower triangular heatmap. The color scale represents Spearman’s correlation coefficients (ρ), ranging from −1 (strong negative correlation, blue) to +1 (strong positive correlation, red). OLEC: olive leaf extract (ultrasound-assisted) from Coratina; OLIC: olive leaf infusion from Coratina; OLEP: olive leaf extract (ultrasound-assisted) from Picual; OLIP: olive leaf infusion from Picual.

The antioxidant activity of olive leaf extracts varied according to both the cultivar and extraction method, as well as depending on the assay (Figure 5A). For the ABTS assay, the highest activity was observed in OLIP (222.50 ± 0.19), followed by OLIC (192.00 ± 0.25), OLEC (183.50 ± 0.16), and OLEP (181.10 ± 0.31). DPPH results, on the other hand, showed a different pattern. OLEP (264.50 ± 0.19) and OLIP (260.00 ± 0.37) had higher values, while OLEC (194.40 ± 0.33) and OLIC (170.90 ± 0.40) had lower values. These differences suggest distinct antioxidant behaviors depending on the assay, likely reflecting variations in the chemical composition associated with the Picual, previous analysis.

Correlation analysis (Figure 5B) further supported this interpretation. Specific compounds, particularly flavonoids and secoiridoid derivatives, indicated a tendency to show positive associations with radical scavenging capacity, suggesting their contribution to the antioxidant potential of the extracts. However, these correlations should be interpreted with caution, as they are based on a limited number of experimental conditions (four extract groups: OLEC, OLEP, OLIC, and OLIP).

Compounds such as luteolin, luteolin-7-O-glucoside, luteolin-7-O-rutinoside, and oleuropein derivatives, such as oleuropein aglycone and demethyloleuropein, showed moderate to strong positive correlations with the ABTS and DPPH assays, suggesting a significant contribution to the antioxidant capacity of the extracts. While simple phenolic compounds and flavonoids, including caffeic acid, *p*-coumaric acid, epigallocatechin, and apigenin, showed more moderate or variable positive correlations, this indicates that they may play a complementary role in antioxidant activity. In contrast, compounds such as Vicenin II and some specific glycosylated derivatives exhibited weak or negative correlations, suggesting a lesser individual contribution. These associations may occur due to the relationship between chemical structure and antioxidant activity, where compounds containing catechol groups and a higher number of hydroxyls, such as caffeic acid and oleuropein derivatives, exhibit a greater capacity to neutralize free radicals [28,38].

Furthermore, clusters of molecules with similar patterns were found in the Spearman correlation matrix, particularly among oleuropein derivatives and glycosylated flavonoids, suggesting potential synergistic effects on antioxidant activity. This result implies that the interaction between the many phenolic classes found in the extracts is what determines the observed antioxidant capacity rather than a single molecule. The antioxidant activity and other bioactive activities of the compounds found in olive leaves have been shown to occur from both the individual actions of the compounds and the synergistic effects of several phenolic classes, which supports our findings [14,19,67]. The clustering in the correlation matrix aligns with the idea that the bioactivity of the extracts depends on the interaction between their constituents. However, it should be considered that the magnitude of these associations may vary depending on the analytical method used, as assays like ABTS and DPPH involve different mechanisms of action [40,68].

In line with these results, Zhang et al. (2022) showed that levels of glycosylated flavonoids, such as luteolin 7-O-glucoside, kaempferol 7-O-glucoside, and rutin, as well as secoiridoids, such as oleuropein, secologanin, and hydroxytyrosol, are correlated with antioxidant activity as determined by ABTS and FRAP [13]. Positive correlations with several substances as opposed to only one particular marker were shown by Spearman correlation coefficients. According to Lorini et al. (2020), the correlation analysis, which was also carried out in Brazilian cultivars, showed total phenolic content had the strongest link with antioxidant activity as determined by FRAP, ABTS, and DPPH. On the other hand, while being the most common class of chemicals in the extracts, flavonoid levels only moderately correlated with antioxidant activity [38].

Additionally, descriptive associations further support the observed correlations. Preczenhak et al. (2025) highlighted that antioxidant activity is influenced not only by the presence of intact compounds but also by the formation of degradation by-products [48]. They reported lower ABTS antioxidant activity following the boiling process in infusions of Brazilian olive leaves. A similar trend was observed in our results using the ABTS assay. Similarly, Nicolì et al. (2019), in the evaluation of 15 cultivars, reported that Itrana, Apollo, and Maurino exhibited the highest total phenolic content, consistent with their elevated antioxidant activity [12]. This pattern is in agreement with our findings, particularly for OLEC and OLEP as assessed by the DPPH assay. Further supporting the proposed synergistic interactions among compounds, Palmeri et al. (2022), when assessing antioxidant activity by DPPH and ORAC, attributed the observed effects to the overall metabolic profile rather than to individual constituents [15].

Thus, our results are consistent with previous studies demonstrating that olive leaf extracts are rich in phenolic compounds, particularly those derived from oleuropein-related metabolites and flavonoids such as luteolin and its glycosides, which are directly associated with antioxidant activity [38,65]. Moreover, secondary and minor compounds have been suggested as key modulators of antioxidant activity, rather than the most abundant constituents [13,15]. Overall, the observed patterns contribute to a better understanding of the relationship between the metabolic profile and bioactivity, particularly in terms of antioxidant activity.

## 3. Materials and Methods

### 3.1. Aqueous Extraction Procedures

#### 3.1.1. Olive Leaf Acquisition

Olive leaves from the Picual and Coratina varieties, cultivated in Brazil’s Serra da Mantiqueira region (21°45′52.39″ S, 46°39′37.29″ W), were directly sourced from the farmers. To obtain extracts from the Brazilian olive leaves of each cultivar, two distinct aqueous extraction methods were employed. The leaves were dehydrated at 120 °C for 15 min in a vented oven (Model 330, Fanem, São Paulo, Brazil) to attain a moisture content of less than 1%, followed by grinding with a blender, according to Difonzo et al. (2017) and Pessoa et al. (2026) [27,39].

#### 3.1.2. Ultrasound-Assisted Extraction (UAE)

The olive leaf extract was obtained by ultrasound (OLE) following the method described by Difonzo et al. (2017); water was added to the ground leaves (at ratios of 1:20, *w*/*v*) and subjected to an ultrasonic bath three times, each for 30 min at room temperature (yielding 60 mL/g of sample). Finally, the extracts obtained were filtered through filter paper (67 g/m^2^) [27].

#### 3.1.3. Aqueous Infusion Extraction (AIE)

The aqueous infusion extraction was conducted following the methodology delineated by Ramírez et al. (2023). Briefly, 1.7 g of dried and ground leaves were infused in 240 mL of boiling water for 5 min, with occasional stirring (solid-to-liquid ratio of 1:140, *w*/*v*). The resulting extracts were then filtered through filter paper (67 g/m^2^) [31].

### 3.2. Analysis Methods

#### 3.2.1. Phenolic Metabolomic Profiling by UHPLC-DAD-MS/MS

Tandem mass spectrometry analysis by ultra-high performance liquid chromatography with electrospray ionization (UHPLC-DAD-MS/MS) (Thermo Fischer Scientific, Waltham, MA, USA) was performed according to Difonzo et al. (2017) and Pessoa et al. (2026) with minor adaptations [27,39]. The system consisted of a UHPLC Dionex Ultimate 3000 (LPG-3400 RS quaternary pump, WPS-3000 TRS autosampler, TCC-3000 RS column oven and photodiode array detector, PDA), coupled to a HESI-II ion source (Thermo Fisher Scientific) and the LTQ Velos Pro linear ion trap mass spectrometer (Thermo Fischer Scientific, Waltham, MA, USA). The PDA detector was set to scan from 220 to 600 nm of wavelength managed by a 3D field.

Phenolic compounds were separated on a Hypersil Gold aQ C18 column (1.9 μm, 2.1 × 100 mm) maintained at 30 °C, using a mobile phase composed of (A) water/formic acid (99.9:0.1, *v*/*v*) and (B) methanol/acetonitrile/formic acid (94.9:5:0.1, *v*/*v*/*v*) at a flow rate of 0.3 mL/min. The gradient elution followed: 0–1 min, 95% A; 1–18 min, 55% A; 18–21 min, 65% A, 21–25 min, 95% A (re-equilibration phase). UV detection was monitored at 280 nm to support phenolic profiling.

The MS conditions were capillary temperature 320 °C; source heater temperature 280 °C; nebulizer gas N2; sheath gas flow 30 psi; auxiliary gas flow 7 arbitrary units; capillary voltage −2800 V, S-Lens RF Level 60%. Mass spectrometric data were acquired in negative ionization mode, enabling the detection of deprotonated molecular ions [M–H]^−^. A full scan method (*m*/*z* 100–900) was applied for compound screening and semi-quantitative estimation based on extracted ion chromatograms (EICs) and peak area (AUC) integration.

Phenolic compound identification attempts were made by combining retention times, accurate molecular ions, MS/MS fragmentation patterns, and literature data. Fragmentation patterns were obtained via data-dependent acquisition (DDA). The DDA parameters included a scan range of *m*/*z* 250–1200, activation threshold of 65,000 counts, isolation width of 2 Da, and collision-induced dissociation (CID) energy of 35. Authentic reference standards were not used for confirmation of all individual phenolic compounds; therefore, the reported compounds were considered tentatively identified/annotated based on the available analytical evidence.

Semi-quantitative estimation was performed by molecular ion extraction in post-acquisition, providing semi-quantitative estimates based on peak areas relative to a single external standard, luteolin-7-glucoside. A calibration curve was established using luteolin-7-glucoside as a single external standard in the concentration range of 0.1–5 μg/mL. Results are expressed as luteolin-7-glucoside equivalents (mg/100 g dry weight of leaves). All analyses were performed in triplicate.

#### 3.2.2. DPPH and ABTS Radical Scavenging Assays

The antioxidant capacity of the extracts and infusions was evaluated using ABTS and DPPH radical scavenging assays.

The ABTS assay was based on the ability of the samples to quench the ABTS radical cation (ABTS·+), using Trolox as a reference standard. The ABTS·+ radical was generated by reacting ABTS (7 mM in distilled water) with potassium persulfate (K_2_S_2_O_8_, 140 mM), and the mixture was kept in the dark at room temperature for 12–16 h to allow complete radical formation. Prior to analysis, the solution was diluted with ethanol to an absorbance of 0.70 ± 0.02 at 734 nm. The assay was carried out in spectrophotometric cuvettes by mixing 50 μL of sample with 950 μL of the ABTS·+ solution, and the decrease in absorbance was measured at 734 nm after 8 min.

The DPPH assay was based on the ability of the samples to scavenge the stable DPPH radical (2,2-diphenyl-1-picrylhydrazyl), using Trolox as a reference standard. A DPPH solution in ethanol was prepared with an initial absorbance of 0.80 ± 0.02 at 517 nm. In cuvettes, 50 μL of each sample were added to 950 μL of the DPPH solution and kept in the dark for 30 min. The decrease in absorbance was then recorded at 517 nm.

All measurements were performed using a Cary 60 UV-Vis spectrophotometer (Agilent Technologies, Milan, Italy). Results were expressed as μmol Trolox equivalents (TE) per gram of dry weight of the extracts. All experiments were performed in triplicate (*n* = 3), and each measurement was carried out in technical triplicate.

#### 3.2.3. Statistical Analysis

Multivariate statistical analyses were performed using R software (version 4.5.3) to investigate variations in phenolic metabolite profiles among samples. Prior to analysis, data were autoscaled by mean-centering and dividing by the standard deviation. Principal Component Analysis (PCA) was applied to evaluate sample clustering and discrimination according to extraction method and cultivar. The number of principal components retained was based on explained variance. Hierarchical clustering analysis was conducted to generate heatmaps representing metabolite abundance patterns across samples. Clustering was performed using Euclidean distance and the complete linkage method for both metabolites and samples. Spearman’s rank correlation analysis was used to assess associations between phenolic compounds and antioxidant activity. Correlation coefficients (ρ) were calculated, and results were visualized as a lower triangular correlation matrix.

For antioxidant activity assays, data are expressed as mean ± standard deviation (SD) of three independent experiments. Statistical differences among groups were evaluated using one-way analysis of variance (ANOVA) followed by Tukey’s multiple comparisons test, with significance set at *p* < 0.05. These analyses were performed using GraphPad Prism software (version 8.0.1).

Graphical visualizations, including PCA plots, heatmaps, and RT–*m*/*z* scatter plots, were generated using the ggplot2, pheatmap, and EnhancedVolcano packages in R.

## 4. Conclusions

This study aimed to characterize the phenolic metabolome and antioxidant activity of olive leaves from two cultivars grown in Brazil (Coratina and Picual), evaluating two extraction methods: ultrasound-assisted extraction and infusions. Results show that olive leaves from Brazilian cultivars have a very complex phenolic profile that is primarily made up of secoiridoids. The most prevalent metabolite in all samples was oleoside, which was followed by elenolic acid glycoside. Oleuropein and 10-hydroxyoleuropein were also consistently present, indicating a central core marked by metabolites derived from oleuropein. Flavonoids were also found, although primarily as secondary chemicals whose presence in the composition is greatly influenced by the cultivar and the extraction technique. Luteolin glucoside, which was linked to olive leaf extracts, was highlighted.

The employed metabolomic approach, utilizing UHPLC-DAD-MS/MS and multivariate analyses, facilitated a thorough characterization, surpassing conventional chromatographic methods by elucidating the critical role of medium and low abundance metabolites in sample discrimination. Among these, 10-hydroxyoleuropein, luteolin glucoside, apigenin-O-rutinoside, caffeoylmalic acid, and hydroxytyrosol glucoside showed marked differences among extraction procedures and/or cultivars, contributing to the differentiation of the metabolic profiles.

The two extraction approaches resulted in distinct phenolic profiles and antioxidant responses under the experimental conditions evaluated. Ultrasound-assisted extraction resulted in higher recovery of several phenolic compounds, whereas the antioxidant responses varied according to the assay. In the ABTS assay, the infusions showed higher antioxidant activity than the corresponding ultrasound-assisted extracts, while the DPPH assay showed a different response pattern. The infusions, although generally less concentrated in phenolic compounds, retained a phenolic profile characterized by secoiridoids and showed relevant antioxidant activity under the conditions evaluated. Considering that infusion is a simple and commonly used household preparation method, these findings suggest its potential as a practical approach for obtaining phenolic compounds from olive leaves for dietary use, although further studies involving gastrointestinal digestion, sensory evaluation, and human studies are needed to assess their biological relevance and health effects. Furthermore, the antioxidant activity was consistently linked to various chemicals, emphasizing that the bioactive impact is determined by synergistic interactions within the entire metabolic profile, rather than by isolated individual indicators.

This study establishes an unprecedented metabolomic foundation for Brazilian-grown olive leaf cultivars and illustrates that extraction methods affect both the bioactive composition and in vitro antioxidant activity of this agro-industrial by-product, thereby broadening its application possibilities in functional foods or beverages and promoting sustainable waste utilization.

## Figures and Tables

**Table 1:** Detailed profiling of phenolic metabolites identified in OLE by UHPLC-DAD-MS/MS.

tR (min)	[M-H]^−^ (*m*/*z*)	Compound	λmax (nm)	Main MS/MS	Ref.
Olive leaf extracts-Coratina cultivar (OLEC)					
1.65	295.26	Caffeoylmalic acid	292	179.01/131.06/87.10	[5,21]
1.79	685.21	Oleuropein-O-deoxyhexoside	280	539.05	[40]
4.33	389.36	Oleoside (1)	282	165.08/208.95/345.04/227.01/121.02/182.98	[34,40]
5.7	315.28	Hydroxytyrosol glucoside	280	153.02/123.05/135.02	[27,34,40]
6.16	375.29	Loganic acid (1)	280	331.08	[40]
7.22	375.3	Loganic acid (2)	256	194.99/151.11/331.09	[40]
7.37	447.18	Luteolin-O-hexoside	284–316	151.96	[40]
8.71	435.24	Phloridzin	282	346.96/272.98	
9.39	305.13	Epigallocatechin (1)	-	225.04/96.94/245.06	[40]
9.7	505.36	*p*-Coumaric acid diglucoside	324	160.98/178.98/280.99/250.97/220.98	[41]
10.43	389.18	Oleoside (2)	234	345.01	[34,40]
10.67	403.24	Elenolic acid glucoside	-	223.01/179.01/371.04	[34,40]
11.29	307.21	Epigallocatechin (2)	-	225.07/96.99	[42,43]
12.24	377.16	Oleuropein aglycon	280–324	197.03/153.06	[44]
13.88	623,19	Verbascoside	-	557,1/461,7	[27,34,40]
13.91	593.14	Vicenin II	274–330	473/352.97/503.02	[34]
14.58	555.26	10-Hydroxyoleuropein (1)	-	537.09/403.06/323.03/223.01	[27,34,40]
15.43	609.22	Luteolin diglucoside	270–330	447.02	[34,40]
17.11	555.22	10-Hydroxyoleuropein (2)	278	537.05	[27,34,40]
17.83	447.21	Luteolin glucoside (1)	344	284.98	[27,34,40]
18.18	593.33	Luteolin rutinoside	278	547.15/284.96	[34,40]
18.35	609.23	Rutin	336	300.97	[40,43]
18.49	539.19	Oleuropein (1)	282–328	337/307/274.98/345	[27,34,40]
19.37	577.25	Apigenin-rutinoside	330	269	[34,40]
19.83	447.15	Quercetin rhamnoside	338	300.96	[40,43]
20.12	447.21	Luteolin glucoside (2)	270–336	285.01	[27,34,40]
Olive leaf extracts-Picual cultivar (OLEP)					
1.66	295.27	Caffeoylmalic acid	292	178.97/131/87.14	[5,21]
1.79	685.25	Oleuropein-O-deoxyhexoside	278	539.05	[40]
4.38	389.3	Oleoside (1)	282	165/208.95/345.01/226.96/120.98/183.97	[34,40]
5.33	389.38	Oleoside (2)	312	208.98/180.98/165/121	[34,40]
5.75	315.24	Hydroxytyrosol glucoside	278	153.01	[27,34,40]
6.29	389.17	Oleoside (3)	280	226.95/183.02/165/121.12/209.05	[34,40]
8.74	435.24	Phloridzin	282	346.97/272.94	[43]
8.88	375.25	Loganic acid	284–386	329.09	[40]
9.42	305.11	Epigallocatechin (1)	-	225.02/96.98	[40]
9.72	505.19	*p*-Coumaric acid diglucoside	240	160.94/179.08/280.94/250.95/221.02	[41]
10.47	389.15	Oleoside (4)	234	345.02	[34,40]
10.66	403.12	Elenolic acid glucoside	236	222.99/179.06/371.03	[34,40]
11.32	307.20	Epigallocatechin (2)	300	225.03/96.94	[42,43]
12.27	377.16	Oleuropein aglycon	300–324	197.03/153.05	[44]
13.93	593.22	Vicenin II	274–328	473/352.98/502.98	[34]
14.13	285.22	Luteolin	290	240.98/199.02/175.01/242.98/217.01	[40]
14.64	555.22	10-Hydroxyoleuropein (1)	282	537.05/403.02/392.99/323.06/223.06	[27,34,40]
15.47	555.23	10-Hydroxyoleuropein (2)	270–334	537.07/393.03/403.04/323/223	[27,34,40]
16.36	555.26	10-Hydroxyoleuropein (3)	286–328	537.02/403.03/323.04/223.09	[27,34,40]
17.25	571.21	Oleuropeinic acid	282	537.09	[40]
17.87	447.87	Luteolin glucoside	348	284.96	[27,34,40]
17.98	565.19	Elenolic acid dihexoside	344	403.01	[40]
18.04	593.19	Luteolin rutinoside (1)	344	284.98	[34,40]
18.2	593.2	Luteolin rutinoside (2)	278	284.94	[34,40]
18.37	701.25	Oleuropein diglucoside	312–336	539.09	[34,40]
18.41	701.27	Oleuropein-O-hexoside	286–336	539.08/377.05/307.02/275.03	[34,40]
18.49	539.19	Oleuropein (1)	282–328	377/307/274.98/345	[27,34,40]
19.41	577.16	Apigenin-rutinoside	268–336	268.96	[34,40]
19.86	539.23	Oleuropein (2)	324	377.04/307.02/275.02/345.04	[27,34,40]
20.16	447.12	Luteolin glucoside (2)	270–336	284.97	[27,34,40]

tR: retention time; [M–H]^−^ (*m*/*z*): deprotonated molecular ion detected in negative ion mode; λmax: maximum absorption wavelength; Main MS/MS: main fragment ions obtained by tandem mass spectrometry. OLEC: olive leaf extract (ultrasound-assisted) from Coratina; OLEP: olive leaf extract (ultrasound-assisted) from Picual. Ref.: References.

**Table 2:** Detailed profiling of phenolic metabolites identified in OLI by UHPLC-DAD-MS/MS.

tR (min)	[M-H]^−^ (*m*/*z*)	Compound	λmax (nm)	Main MS/MS	Ref.
Olive leaf infusion-Coratina cultivar (OLIC)					
1.65	295.29	Caffeoylmalic acid	292	276.97/179.01/131.06/87.10	[5,21]
1.77	685.25	Oleuropein-O-deoxyhexoside	278	539.04	[40]
5.3	389.29	Oleoside (1)	292–314	208.94/180.99/164.99	[34,40]
5.8	315.16	Hydroxytyrosol glucoside	228	152.98/123	[27,34,40]
5.99	389.15	Oleoside (2)	286–360	208.94/121.01/165.06/345.02	[34,40]
6.26	389.13	Oleoside (3)	230	227/183.01/165/121.02/208.97	[34,40]
8.73	435.18	Phloridzin	282	272.98	[43]
9.11	389.22	Oleoside (4)	288	345.05	[34,40]
9.4	305.13	Epigallocatechin (1)	288	225.04/96.94/245.06	[40]
9.7	505.19	*p*-Coumaric acid diglucoside	284	161.03/178.96/280.92/250.95/220.97	[41]
10.44	389.13	Oleoside (5)	234	345.05/208.98/165.05/121.02/183.02	[34,40]
10.64	403.17	Elenolic acid glucoside (1)	234	222.97/179.08/371.02	[34,40]
11.29	307.21	Epigallocatechin (2)	296	221.07/96.99	[42,43]
12.24	377.17	Oleuropein aglycon	292–324	197.01/153.04	[44]
12.77	375.21	Loganic acid	288–330	215.01	[40]
13.6	403.23	Elenolic acid glucoside (2)	-	371/222.98/179	[34,40]
14.61	555.15	10-Hydroxyoleuropein (1)	222–282	537.03/403/393.02/323/222.98	[27,34,40]
14.97	525.2	Demethyl-oleuropein	284	195.07/319.02/363.03/249	[34,40]
15.31	555.27	10-Hydroxyoleuropein (2)	258–280	537.01/393.02/402.99/322.95	[27,34,40]
17.81	447.19	Luteolin glucoside (1)	252–348	284.94	[27,34,40]
18.18	593.33	Luteolin rutinoside	278	547.15/284.96	[34,40]
18.45	539.16	Oleuropein (1)	220–284	377.08/307.04/275.05/345.04	[27,34,40]
19.36	577.26	Apigenin-rutinoside	336	268.96	[34,40]
19.81	539.24	Oleuropein (2)	344	377.06/307.01/275.05/345.05	[27,34,40]
20.1	447.15	Luteolin glucoside (2)	270–338	284.94	[27,34,40]
Olive leaf infusion-Picual cultivar (OLIP)					
1.65	295.34	Caffeoylmalic acid	292	87.14/179/131.08	[5,21]
1.79	685.21	Oleuropein-O-deoxyhexoside	280	539	[40]
5.3	389.29	Oleoside (1)	292–314	208.94/180.99/164.99/121.06	[34,40]
5.8	315.16	Hydroxytyrosol glucoside	228	152.98/123/135.06	[27,34,40]
5.99	389.15	Oleoside (2)	286–360	208.94/121.01/165.06/139.09/345.02	[34,40]
6.26	389.13	Oleoside (3)	230	227/183.01/165/121/208.97/139.15/345.11	[34,40]
7.3	375.28	Loganic acid	284	329.08/151.01	[40]
8.73	435.18	Phloridzin	282	272.98	[43]
9.11	389.22	Oleoside (4)	288	345.05	[34,40]
9.4	305.13	Epigallocatechin (1)	288	225.04/96.94/245.06	[40]
9.7	505.19	*p*-Coumaric aciddiglucoside	284–332	161.03/178.96/280.92/250.95/220.95	[41]
10.44	389.16	Oleoside (5)	234–338	345.04/209.02/165.01/121.04/183.1	[34,40]
10.64	403.17	Elenolic acid glucoside (1)	234	222.97/179.08/371.02	[34,40]
11.29	307.21	Epigallocatechin (2)	-	221.07/96.99	[42,43]
12.24	377.17	Oleuropein aglycon	292–322	197.01/153.04	[44]
13.6	403.16	Elenolic acid glucoside (2)	282	371.05/223/179.02	[34,40]
13.89	593.18	Vicenin II	282–336	473/353/503.02	[34]
13.91	623,2	Verbascoside	-	557,09/461,9	[27,34,40]
14.61	555.15	10-Hydroxyoleuropein (1)	222–282	537.1/403.04/232.04/223	[27,34,40]
14.97	525.2	Demethyl-oleuropein	284	319.02/363.03/249	[34,40]
15.31	555.27	10-Hydroxyoleuropein (2)	280	537.01/393.02/322.95/223	[27,34,40]
16.01	555.23	10-Hydroxyoleuropein (3)	284–330	537.08/403.05/223/323.02	[27,34,40]
16.31	555.24	10-Hydroxyoleuropein (4)	288–332	537.03/403.01/222.98/322.98	[27,34,40]
17.19	571.17	Oleuropeinic acid	284	537.07	[40]
17.81	447.19	Luteolin glucoside (1)	252–348	284.94	[27,34,40]
17.98	567.19	Elenolic acid dihexoside	344	403.01	[40]
18.16	593.17	Luteolin rutinoside	280	284.95	[34,40]
18.31	701.27	Oleuropein-O-hexoside	286–336	539.08/377.05/307.02/275.03	[34,40]
18.45	539.24	Oleuropein (1)	284	377.02/306.98/274.99/345.01	[27,34,40]
18.75	701.26	Oleuropein diglucoside	284	539.09	[34,40]
19.36	577.26	Apigenin-rutinoside	336	268.96	[34,40]
19.81	539.24	Oleuropein (1)	344	377.06/307.01/275.05/345.05	[27,34,40]
20.1	447.15	Luteolin glucoside (1)	270–338	284.94	[27,34,40]

tR: retention time; [M–H]^−^ (*m*/*z*): deprotonated molecular ion detected in negative ion mode; λmax: maximum absorption wavelength; Main MS/MS: main fragment ions obtained by tandem mass spectrometry. OLIC: olive leaf infusion from Coratina; OLIP: olive leaf infusion from Picual.

**Table 3:** Semi-quantitative estimation of phenolic metabolites in OLE and OLI from two cultivars. Values are expressed as mg luteolin-7-glucoside equivalents/100 g dry weight (mean ± SD, *n* = 3), based on peak area (AUC).

N°	Class	Compound	Chemical Formula	OLEC	OLIC	OLEP	OLIP
**1**	Secoiridoid	10-Hydroxyoleuropein	C_25_H_32_O_14_	95.52 ± 0.01	44.86 ± 0.06	84.66 ± 0.01	44.66 ± 0.01
**2**	Flavonoid	Apigenin-O-rutinoside	C_27_H_30_O_14_	27.54 ± 0.01	14.70 ± 0.01	58.56 ± 0.01	18.79 ± 0.01
**3**	Hydroxycinnamic acid derivative	Caffeoylmalic acid	C_13_H_12_O_9_	74.70 ± 0.01	8.76 ± 0.02	26.94 ± 0.01	14.09 ± 0.01
**4**	Secoiridoid	Demethyl-oleuropein	C_24_H_30_O_13_	NA	1.36 ± 0.01	NA	0.29 ± 0.01
**5**	Secoiridoid	Elenolic acid dihexoside	C_23_H_34_O_15_	NA	NA	18.90 ± 0.01	10.69 ± 0.32
**6**	Secoiridoid	Elenolic acid glucoside	C_17_H_24_O_10_	122.52 ± 0.01	100.86 ± 0.01	156.96 ± 0.22	101.08 ± 0.06
**7**	Flavanol	Epigallocatechin	C_15_H_14_O_7_	11.10 ± 0.01	2.67 ± 0.01	8.76 ± 0.01	5.81 ± 0.02
**8**	Phenylethanoid glycoside	Hydroxytyrosol glucoside	C_14_H_20_O_8_	12.06 ± 0.01	19.80 ± 0.6	13.26 ± 0.11	21.26 ± 0.01
**9**	Secoiridoid	Loganic acid	C_16_H_24_O_10_	6.96 ± 0.01	1.28 ± 0.01	3.42 ± 0.01	0.99 ± 0.06
**10**	Flavonoid (aglycone)	Luteolin	C_15_H_10_O_6_	NA	NA	8.94 ± 0.01	NA
**11**	Flavonoid	Luteolin-dihexoside	C_27_H_30_O_16_	10.02 ± 0.01	NA	NA	NA
**12**	Flavonoid	Luteolin Glucoside	C_21_H_20_O_11_	64.90 ± 0.2	29.80 ± 0.5	125.30 ± 0.1	29.70 ± 0.01
**13**	Flavonoid	Luteolin-O-hexoside (isomer)	C_21_H_20_O_11_	4.02 ± 0.06	NA	NA	NA
**14**	Flavonoid	Luteolin-O-rutinoside	C_27_H_30_O_15_	9.30 ± 0.01	4.50 ± 0.4	33.42 ± 0.01	4.38 ± 0.02
**15**	Secoiridoid	Oleoside	C_16_H_22_O_11_	861.01 ± 1.27	512.42 ± 1.11	853.63 ± 1.21	500.21 ± 1.13
**16**	Secoiridoid	Oleuropein	C_25_H_32_O_13_	117.66 ± 0.01	87.15 ± 0.06	104.41 ± 0.7	86.28 ± 0.01
**17**	Secoiridoid	Oleuropein aglycone	C_19_H_22_O_8_	14.16 ± 0.01	7.16 ± 0.01	15.84 ± 0.01	6.84 ± 0.01
**18**	Secoiridoid	Oleuropein-di-O-hexoside	C_31_H_42_O_18_	NA	7.31 ± 0.01	8.52 ± 0.01	NA
**19**	Secoiridoid	Oleuropeinic acid	C_25_H_32_O_12_	NA	3.97 ± 0.03	9.18 ± 0.01	NA
**20**	Secoiridoid	Oleuropein-O-deoxyhexoside	C_25_H_32_O_12_	13.14 ± 0.01	2.99 ± 0.01	6.12 ± 0.01	6.35 ± 0.01
**21**	Secoiridoid	Oleuropein-O-hexoside	C_31_H_42_O_13_	NA	NA	17.82 ± 0.01	NA
**22**	Hydroxycinnamic acid derivative	*p*-Coumaric acid diglucoside	C_21_H_28_O_14_	20.34 ± 0.01	1.74 ± 0.01	19.62 ± 0.02	9.28 ± 0.02
**23**	Dihydrochalcone	Phloridzin	C_21_H_24_O_10_	19.08 ± 0.01	1.48 ± 0.01	9.72 ± 0.01	2.23 ± 0.08
**24**	Flavonoid	Quercetin-O-rhamnoside	C_21_H_20_O_11_	26.46 ± 0.01	NA	NA	NA
**25**	Flavonoid	Rutin	C_27_H_30_O_16_	10.38 ± 0.01	8.18 ± 0.01	NA	NA
**26**	Phenylethanoid glycoside	Verbascoside	C_29_H_36_O_15_	8.46 ± 0.01	NA	NA	2.93 ± 0.01
**27**	Flavonoid (C-glycosylated)	Vicenin II	C_27_H_30_O_15_	11.82 ± 0.01	NA	9.54 ± 0.01	2.84 ± 0.01
	Total (mg/100 g dried leaves)			1552.00 ± 2.17	861.00 ± 1.59	1593.80 ± 1.73	868.60 ± 1.85

OLEC: olive leaf extract (ultrasound-assisted) from Coratina; OLIC: olive leaf infusion from Coratina; OLEP: olive leaf extract (ultrasound-assisted) from Picual; OLIP: olive leaf infusion from Picual; NA: not available.

## Data Availability

The data presented in this study are available from the corresponding authors upon reasonable request.

## Supplementary Materials

The following supporting information can be downloaded at: https://www.mdpi.com/article/10.3390/molecules31203740/s1, Figure S1: PCA model validation based on cumulative R^2^X and Q^2^X values across the principal components of the phenolic metabolite dataset.
