# Supplementary material for: Metabolomic Profiling of Phenolic Compounds in Brazilian-Grown Olive Leaf Infusions and Ultrasound-Assisted Extracts
Source: Molecules. 2026 Oct 18;31(20):3740. doi: 10.3390/molecules31203740 (PMC13707651; doi:10.3390/molecules31203740)
Supplement: Supplementary file 1 [file molecules-31-03740-s001.zip › molecules-4591305-supplementary.pdf]

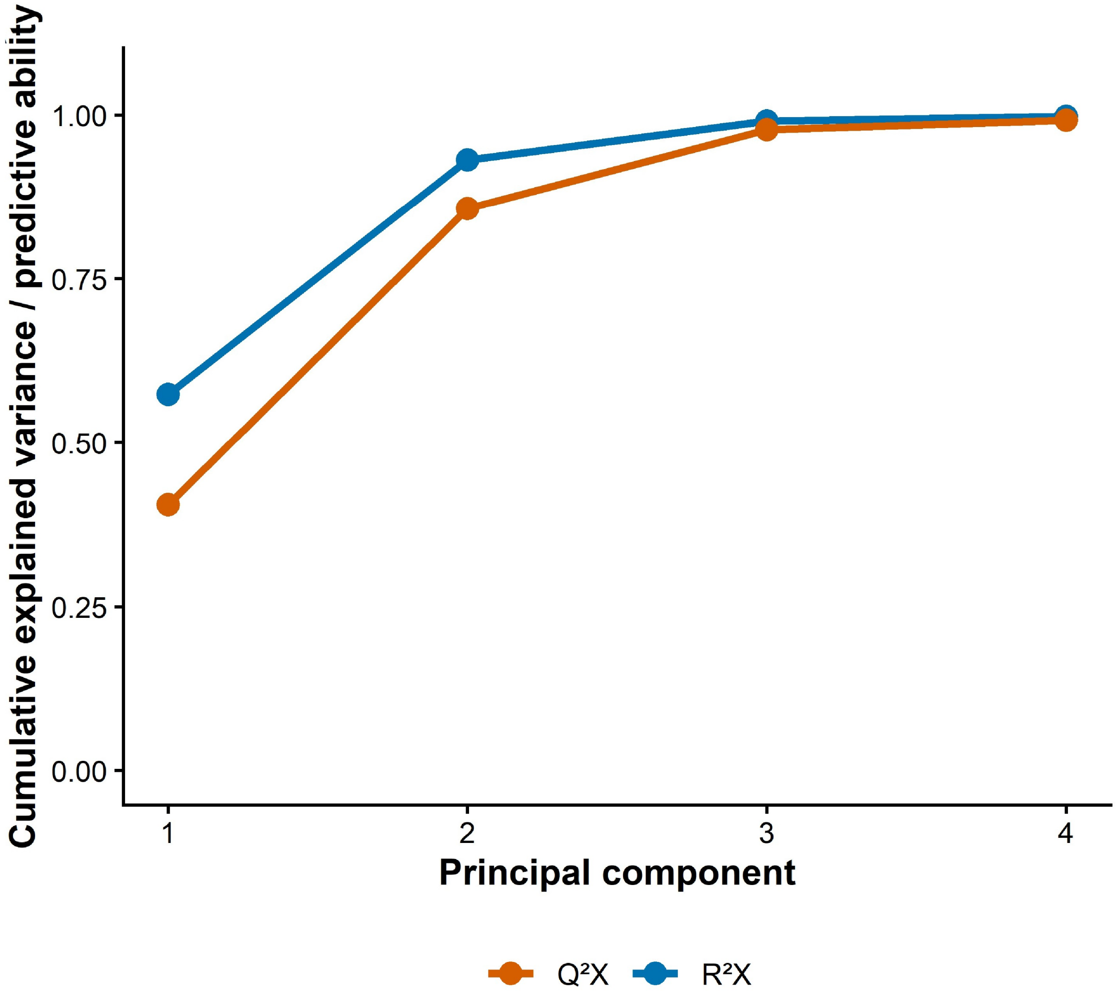

Supplementary Figure S1. PCA model validation based on cumulative R^2X and Q^2X values across the principal components of the phenolic metabolite dataset.
